# Phosphorylated CRMP1, axon guidance protein, is a component of spheroids and is involved in axonal pathology in amyotrophic lateral sclerosis

**DOI:** 10.3389/fneur.2022.994676

**Published:** 2022-09-27

**Authors:** Yuko Kawamoto, Mikiko Tada, Tetsuya Asano, Haruko Nakamura, Aoi Jitsuki-Takahashi, Hiroko Makihara, Shun Kubota, Shunta Hashiguchi, Misako Kunii, Toshio Ohshima, Yoshio Goshima, Hideyuki Takeuchi, Hiroshi Doi, Fumio Nakamura, Fumiaki Tanaka

**Affiliations:** ^1^Department of Neurology and Stroke Medicine, Yokohama City University Graduate School of Medicine, Yokohama, Japan; ^2^Department of Molecular Pharmacology and Neurobiology, Yokohama City University Graduate School of Medicine, Yokohama, Japan; ^3^Department of Biochemistry, School of Medicine, Tokyo Women's Medical University, Tokyo, Japan; ^4^Department of Nursing Course Biological Science and Nursing, Yokohama City University Graduate School of Medicine, Yokohama, Japan; ^5^Department of Life Science and Medical Bioscience, Graduate School of Advanced Science and Engineering, Waseda University, Tokyo, Japan

**Keywords:** ALS, axon guidance, axonopathy, CRMP, motor neuron, neurofilament

## Abstract

In amyotrophic lateral sclerosis (ALS), neurodegeneration is characterized by distal axonopathy that begins at the distal axons, including the neuromuscular junctions, and progresses proximally in a “dying back” manner prior to the degeneration of cell bodies. However, the molecular mechanism for distal axonopathy in ALS has not been fully addressed. Semaphorin 3A (Sema3A), a repulsive axon guidance molecule that phosphorylates collapsin response mediator proteins (CRMPs), is known to be highly expressed in Schwann cells near distal axons in a mouse model of ALS. To clarify the involvement of Sema3A–CRMP signaling in the axonal pathogenesis of ALS, we investigated the expression of phosphorylated CRMP1 (pCRMP1) in the spinal cords of 35 patients with sporadic ALS and seven disease controls. In ALS patients, we found that pCRMP1 accumulated in the proximal axons and co-localized with phosphorylated neurofilaments (pNFs), which are a major protein constituent of spheroids. Interestingly, the pCRMP1:pNF ratio of the fluorescence signal in spheroid immunostaining was inversely correlated with disease duration in 18 evaluable ALS patients, indicating that the accumulation of pCRMP1 may precede that of pNFs in spheroids or promote ALS progression. In addition, overexpression of a phospho-mimicking CRMP1 mutant inhibited axonal outgrowth in Neuro2A cells. Taken together, these results indicate that pCRMP1 may be involved in the pathogenesis of axonopathy in ALS, leading to spheroid formation through the proximal progression of axonopathy.

## Introduction

Amyotrophic lateral sclerosis (ALS) is a fatal and progressive neurodegenerative disorder caused by selective loss of both upper motor neurons in the motor cortex and lower motor neurons in the brain stem and spinal cord (1). Ninety percent of cases with ALS are sporadic and 10% are familial. At least 27 genes have been found to be definitively associated with familial ALS (2). Multiple hypotheses have been proposed regarding the molecular pathogenesis of ALS, such as mitochondrial dysfunction, cytoskeletal impairment, axonal transport dysfunction, toxic protein aggregation, impaired protein degradation, excitotoxicity, decreased neurotropic support from non-neuronal cells, oxidative stress, hypermetabolism, inflammation, RNA metabolism defects, and RNA toxicity (3).

Among these, the importance of cytoskeletal impairment and disrupted axonal transport is directly suggested by the function of proteins encoded by several genes responsible for familial ALS, including neurofilament heavy chain (4), dynactin subunit 1 (5), peripherin (6), profilin 1 (7), tubulin alpha 4a (8), and kinesin family member 5A (9). The involvement of axonal dysfunction is further demonstrated by the fact that previous studies in patients and animal models of ALS have shown that the pathology of motor neuron axons begins at distal sites, including neuromuscular junctions (NMJs), and progresses proximally in a “dying back” manner prior to the degeneration of cell bodies (distal axonopathy) (10–13).

Among the candidate mechanisms of distal axonopathy in ALS is semaphorin-3A (Sema3A) signaling (12). Sema3A is an important repulsive axon guidance cue involved in neurodevelopmental pattern formation (14). Sema3A binds to the neuropilin-1 (NRP1) and plexin A receptor complexes and activates downstream kinases such as cyclin-dependent kinase 5 (Cdk5). Sema3A signaling mediates axon guidance, dendritic outgrowth, and synapse formation *via* the phospho-regulation of downstream proteins, including collapsin response mediator proteins (CRMPs) 1 and 2 (15–19).

In the G93A-SOD1 transgenic mouse model of ALS, Sema3A is upregulated in specific populations of terminal Schwann cells located at the distal ends of motor neuron axons near fast-fatigable muscle fibers that are particularly vulnerable to denervation in ALS (20). In addition, the administration of anti-NRP1 antibody, which blocks Sema3A signaling, improved motor function and survival and reduced denervation of NMJs in G93A-SOD1 mice (21), and similar ameliorative effects are observed after inhibition of CRMP2 phosphorylation (22). Furthermore, we recently reported that while *Crmp1* knockout leads to deterioration of motor function in G93A-SOD1 mice, phospho-null Crmp1 improves motor function and prevents motor neuron loss and denervation of NMJs (23).

These findings suggest that Sema3A–CRMP signaling may be involved in the pathogenesis of ALS, especially in terms of triggering distal axonopathy. However, the CRMP family has not been examined in ALS patient tissues. Because CRMP phosphorylation is critical as the effector of Sema3A signaling, in this study we investigated the status of CRMP1 phosphorylation in the spinal cords of 35 ALS patients. We found that Thr509-phosphorylated CRMP1 (pCRMP1) was co-localized with phosphorylated neurofilaments (pNFs) at the sites of proximal axon swelling (spheroids) in the spinal cord. In ALS patients with short disease duration, the accumulation of pCRMP1 in spheroids was more evident than that of pNFs. In addition, *in vitro* experiments revealed that overexpression of a phospho-mimicking CRMP1 mutant, Thr509Asp-CRMP1, impaired the neurite outgrowth in Neuro2a cells. These findings suggest that pCRMP1 may be involved in the pathogenesis of distal-to-proximal axonal dysfunction in ALS.

## Materials and methods

### *Crmp1* knockout (KO) mice

Details of the generation of *Crmp1* KO mice were described previously (24). The mutant mice were maintained heterozygous on a C57BL/6 background. Three to five mice of the same sex were housed per aluminum cage (W 32.5 cm × D 22.5 cm × H 11 cm) with a floor mat, at a temperature of 21 ± 3°C and humidity of 30–70% on a 12-h light/dark cycle in the animal facility at Yokohama City University Graduate School of Medicine. These mice were fed an autoclaved diet and given water ad libitum. Homozygous mice were obtained by crossing heterozygous mutant mice. Mice were sacrificed by an overdose of isoflurane (7–8%). Four mice were analyzed in this study. This study was approved by the Institutional Review Board of Yokohama City University School of Medicine (FA16-069). All procedures were conducted according to the guidelines of the Institutional Animal Care and Use Committee of the Yokohama City University School of Medicine.

### Antibodies

Antibody against phosphorylated Thr509-CRMP1 (pThr509-CRMP1) was generated as described previously (25). Briefly, rabbits were immunized with a synthetic phosphopeptide [VYEVPA(pT)PKHAAPC: mouse CRMP1 amino acids 503–515 plus cysteine] conjugated to keyhole limpet hemocyanin. The antibody was affinity-purified through a phosphopeptide column. Other antibodies used in this study are listed in Supplementary Table S1.

### Immunoblot analysis of mouse brain lysate

Four-week-old male C57BL/6 and *Crmp1* KO mice were sacrificed as described above. Cerebral cortices from mice were homogenized in immunoprecipitation (IP) buffer containing 20 mM Tris-HCl (pH 8.0), 150 mM NaCl, 1 mM EDTA, 10 mM NaF, 1 mM Na_3_VO_4_, 1% Nonidet P-40, and 50 μM ρ-APMSF (ρ-amidinophenylmethanesulfonyl fluoride). The lysates were centrifuged at 17,600 g for 15 min at 4°C, and supernatants were diluted to a 1 mg/ml concentration. The samples were then used for immunoblotting analysis with anti–pThr509-CRMP1 antibody (1:3,000 dilution).

### Immunohistochemistry of mice brain sections

Ten-week-old male C57BL/6 mice and *Crmp1* KO mice were sacrificed as described above. The mice were then perfused with phosphate-buffered saline [PBS; 137 mM NaCl, 2.68 mM KCl, 8.09 mM Na2HPO4, 1.47 mM KH2PO4 (pH7.4)], followed by 4% paraformaldehyde (PFA). Brains were extracted and incubated in 4% PFA at room temperature for 2 h and then transferred to PBS. Coronal sections (50 μm) were sliced using a Leica VT1200 vibratome. The sections were soaked in 0.3% Triton X-100 in PBS (PBST) containing 0.3% H_2_O_2_ for 30 min and rinsed with PBST. The sections were blocked with PBST−3% normal goat serum (NGS) for 30 min and incubated with anti–pThr509-CRMP1 antibody (1:1,000 dilution with PBST−3% NGS) at 4°C overnight. After rinsing with PBST, the sections were incubated with anti–rabbit IgG secondary antibody–conjugated biotin (Vector Laboratories, Cat No. BA-1000, 1:1,000 dilution with TBST−2% NGS) for 2 h at room temperature. The sections were washed with PBST and immersed in avidin–biotin–peroxidase complex (Vector Laboratories, ABC kit, Cat No. PK-6100) for 1 h at room temperature. The sections were rinsed with PBST and immersed in diaminobenzidine solution (MBL, Histostar, Cat No. 8469) for 10 min. After rinsing with PBST, the sections were mounted onto glass slides.

### Patient samples

The use of autopsied samples was approved by the institutional review board of Yokohama City University School of Medicine (B090903014). Written informed consent was obtained for all experiments involving autopsy tissues.

We pathologically investigated the lumbar spinal cords of 35 sporadic ALS patients (age 70.11 ± 10.81 years) and seven disease control patients (age 79.14 ± 11.13 years) (Table 1) autopsied at Yokohama City University Hospital or Yokohama City University Medical Center. All ALS patients were clinically diagnosed according to the revised El Escorial criteria (26) and the diagnosis was confirmed pathologically. Only one patient did not have obvious upper motor neuron symptoms, but this patient was diagnosed with ALS based on the disease course and the pathological involvement of the corticospinal tract. Four patients with frontotemporal dementia (FTLD) were included in this study (Table 1). Pathologically, the presence of cytoplasmic inclusions positive for phosphorylated 43-kDa transactivating response region DNA–binding protein (TDP-43) was confirmed in all ALS patients.

**Table 1:** Clinical and pathological information in ALS patients and disease controls.

		**ALS**		**Control**	
		**No. of patient**		**No. of patient**	
Diagnosis		ALS	35	Control	7
	Initial symptom site	Bulbar	8	CI	1
		UL	19	CPA	1
		LL	8	AGD	1
	Special note	FTLD	4	HAM	1
		Head trauma	1	Sarcoidosis	1
		Ventilation	2	CCA	1
		UMN (–)	1	AD	1
Sex (male:female)		28:7		2:5	
Walking score (ALSFRS-R)		1	14		
		2	13		
		3	6		
		Unknown	2		
		**Mean** **±SD (range)**		**Mean** **±SD (range)**	
Age at death (years)		70.11 ± 10.81 (41–87)		79.14 ± 11.13 (53–89)	
Disease duration (months)		29.2 ± 26.31 (3–144)		
No. of pCRMP1-positive spheroids		6.06 ± 7.76 (0–40)		1.14 ± 1.36 (0–4)	
No. of residual neuron		26.97 ± 15.25 (1–58)		38 ± 14.27 (12–63)	
Area of anterior horn (mm^2^)		6.27 ± 2.31 (1.42–10.19)		7.26 ± 2.05 (3.07–8.95)	

Initial symptom sites were classified as upper limb (UL), lower limb (LL), and bulbar region. The walking item score in the ALS Functional Rating Scale–Revised (ALSFRS-R) represents the score measured just before death. Notable additional information is presented as a special note, and includes lack of upper motor neuron symptoms (UMN-), use of a ventilator, and comorbidity of frontotemporal lobar degeneration (FTLD) and head trauma. The disease controls include individuals with cerebral infarction (CI), cardiopulmonary arrest (CPA), argyrophilic grain dementia (AGD), human T-cell leukemia virus type 1–associated myelopathy (HAM), sarcoidosis, cortical cerebellar atrophy (CCA), and Alzheimer's disease (AD). The numbers (No.) of pThr509-positive spheroids and residual motor neurons, and the area of the anterior horn, were evaluated in single sections of the lumbar spinal cord (L4 or L5). SD, standard deviation.

### Immunohistopathology of patients' spinal cords

Each lumbar spinal cord was fixed in 20% formalin for 1–2 weeks, embedded in paraffin, and cut into 6-μm sections. Sections from each case were deparaffinized and stained with hematoxylin and eosin, and the Klüver-Barrera method was used to assess the number of neurons.

Staining with 3,3′-diaminobenzidine tetrahydrochloride (DAB) was performed as reported previously (27). After paraffin removal, sections were autoclaved at 121°C for 10 min with 10 mM citric acid (pH 6.0), and then incubated in 1% H_2_O_2_ for 20 min. Sections were then incubated in blocking solution (7% goat serum, 0.1% Triton X-100 in PBS) for 20 min at room temperature. Sections were incubated with anti–pThr509-CRMP1 antibody (1:300) for 2 days at 4°C, washed with PBS, incubated with a biotinylated secondary antibody for 2 h at room temperature, and reacted with avidin–biotin–peroxidase complex (Vectastain ABC kit, Vector Laboratories, Cat No. PK6100). The sections were washed with PBS then incubated with staining solution (0.1% DAB, Dojindo, Cat No. 411496) with or without 0.1% nickel (ammonium nickel sulfate hexahydrate, Wako, Cat No. 146-01012) for 15–20 min at room temperature. Then the sections were counterstained with hematoxylin (Muto Pure Chemicals, Cat No. 30022), dehydrated, and coverslipped.

Peptide blocking was performed as follows. Prior to reacting with tissue sections, the diluted anti–pThr509-CRMP1 antibody solution was preincubated with an equal volume of 1 mg/ml human CRMP1 phosphopeptide [VYEVPA(pT)PKHAAP-c] overnight at 4°C.

For the dephosphorylation assay, sections were incubated with calf intestinal alkaline phosphatase (100 U/ml CIP (New England Biolabs, Cat No. M0290) in 100 mM Tris HCl (pH 8.5) and 1% protease inhibitor cocktail (Nacalai Tesque, Cat No. 03969–21) for 3 h at 37°C prior to immunohistochemistry.

Immunofluorescence histochemistry was performed as follows. Sections were deparaffinized and autoclaved at 121°C for 10 min with 10 mM citric acid (pH 6.0). Lipofuscin Autofluorescence Quencher (TrueBlack, Biotium, Cat No. 23007) was left on the sections for 30 s. Then, the sections were rinsed with PBS and incubated in blocking solution (7% goat serum, 0.1% TritonX-100 in PBS) for 20 min at room temperature. The sections were incubated with the diluted primary antibodies, anti–pThr509-CRMP1 rabbit antibody (1:300), and anti–phosphorylated neurofilament H antibody (mouse monoclonal IgG1, 1:1,000; BioLegend, Cat No. SMI 31P) for 2 days at 4°C. Then the sections were incubated with secondary antibody solution containing Alexa Fluor 488 goat anti–rabbit IgG (H + L) (1:1,000; Thermo Fisher Scientific, Cat No. 11034), Alexa Fluor 568 goat anti–mouse IgG (H + L) (1:1,000; Thermo Fisher Scientific, Cat No. 11019), and Hoechst 33342(1:5,000; Thermo Fisher Scientific, Cat No. H3570) for 2 h at room temperature, protected from light. Coverslips were mounted using ProLong Gold anti-fade reagent (Thermo Fisher Scientific, Cat No. P36934).

With the Bodian silver method, normal neurite and cytoskeleton aggregations (e.g., spheroids) were stained reddish purple with silver protein. Sections were deparaffinized and impregnated with silver in 1% silver protein solution at 37°C overnight. Sections were rinsed with distilled water (DW) and incubated in reducing solution (hydroquinone and anhydrous sodium sulfate solution) for 10 min. Sections were then rinsed in DW and incubated in toning solution (1% gold chloride solution) for 1 h. Next, sections were rinsed in DW and reduced (1% oxalic acid solution) for 5 min. Finally, sections were rinsed in DW and fixed (1% thiosulfuric acid) for 10 min, then dehydrated.

### Quantification and statistical analysis of spheroids in patients' spinal cords

DAB-stained lumbar spinal cord sections from patients with ALS and disease controls were examined using a Nikon scanner under ×40 magnification. To analyze the number of spheroids, pThr509-CRMP1–positive spheroids with a solid brown color were counted in a section of the anterior horn of the lumbar spinal cord. The criterion for defining spheroids was the presence of oval or round structures in the neurons. The lower confidence limit (20 μm) was thereafter used as the cut-off for defining pathologic spheroids. Spheroid diameter was measured using the measurement tool of the NIS-elements D software program (Nikon). Neuronal cell bodies were evaluated by the following criteria: size (>10 μm), shape (triangular or oval), location (cortical layer), and nucleus (round shape). Analysis was performed using the Mann–Whitney *U*-test because of the limited sample size and the non-normal distribution of the number of spheroids in each ALS patient and disease control.

For immunofluorescence analysis of spheroids, slides were imaged using a BZ-X800 microscope (Keyence). pThr509-CRMP1 was visualized with the Alexa Fluor 488 channel, pNFs with the Alexa Fluor 568 channel. Five spheroids were selected per spinal cord section, and the fluorescence intensities of pThr509-CRMP1 and pNFs were measured using ImageJ software in 18 evaluable ALS patients.

First, Spearman's rank-order correlation coefficient was used to assess the degree of association between the duration of ALS and the fluorescence intensities of pThr509-CRMP1 and pNFs in spheroids. Next, patients with ALS were divided equally into three groups [short-term, 7.67 ± 3.08 (3–11) months, *n* = 6; medium-term, 21.3 ± 5.50 (14–30) months, *n* = 6; long-term, 41.0 ± 11.0 (31–61) months, *n* = 6] based on ALS duration, and all fluorescence intensities of pThr509-CRMP1 and pNFs were compared between the groups using the Kruskal–Wallis test at a significance level of *p* < 0.05. If there were significant differences, Dunn's *post hoc* comparison was applied following the Kruskal–Wallis test. GraphPad Prism software (v 8.4.3) was used for calculations and graphics creation.

### DNA, cell culture, and transfection

The expression plasmids for V5-tagged CRMP1 and its phosphorylation-mimicking Thr509Asp mutant CRMP1 were generated as previously described (28) using two primers (5′-CCCAAACATGCAGCTCCTGCTCCTTCTGCC-3′, 5′-ATCAGCTGGCACCTCGTACACGGGGCCATC-3′). Cdk5 and His-tagged p35 cDNA were provided by Dr. Ashok B. Kulkarni (National Institute of Dental and Craniofacial Research, Bethesda, MD). The mutation in all constructs was confirmed by DNA sequencing.

Human embryonic kidney (HEK) 293T cells were cultured in Dulbecco's Modified Eagle Medium (DMEM, Thermo Fisher Scientific, Cat No. 10564011) supplemented with 10% fetal bovine serum (FBS, Biosera, Cat No. FB1285/500), 50 U/ml penicillin, and 50 μg/ml streptomycin (P/S, Nacalai Tesque, Cat No. 0936734). These cells were passaged two times weekly for up to 30 times. HEK 293T cells (1.0 × 10^6^ cells/6-well plate) were transfected with expression vectors of human CRMP1 either with or without V5-tagged Cdk5/p35 using Lipofectamine LTX (Thermo Fisher Scientific, Cat No. 5338100). After 2 days of incubation, the cells were rinsed once with PBS and lysed in 500 μl of IP150 buffer (20 mM Tris-HCl (pH 8.0), 150 mM NaCl, 1 mM EDTA, 10 mM NaF, 1 mM Na3VO4, 1% Nonidet P-40, diluted 1,000-fold in Protease Inhibitor Cocktail [Nacalai Tesque, Cat No. 25955-11)]. The lysates were centrifuged at 10,000 g for 15 min at 4°C. The supernatants were subjected to immunoblot analysis.

Neuro2a cells were purchased from American Type Culture Collection (ATCC). Cells were maintained in DMEM (Thermo Fisher Scientific, Cat No. 10564011) supplemented with 1% P/S (Nacalai Tesque, Cat No. 0936734) and 10% FBS (Biosera, Cat No. FB1285/500). Experiments were performed within the first 20 passages after receipt from ATCC. Micro glass plates (Matsunami, Cat No. CS01813) used for cell culture were coated with 100 μg/ml poly-l-lysine (PLL, Sigma-Aldrich, Cat No. P4832) and incubated for 2 h at room temperature. Excess PLL was rinsed off by washing three times with deionized water. Neuro2A cells were transfected at 2 days *in vitro* (DIV) with either wild-type (WT) CRMP1-V5 or Thr509Asp-CRMP1–V5 using Lipofectamine LTX (Thermo Fisher Scientific, Cat No. 5338100), then differentiated at DIV 3 after the medium was replaced with serum-free medium containing 5 mM N^6^, 2′-O-dibutyryladenosine-3′:5′-cyclic monophosphate sodium salt (Nacalai Tesque, Cat No. 0228961). The cells were fixed at DIV 4 and subjected to immunocytochemistry.

### Immunocytochemistry of cultured cells

Differentiated Neuro2a cells were fixed in 4% PFA 48 h after transfection and 24 h after differentiation. Cells were rinsed with PBS and incubated in blocking buffer (PBST supplemented with 5% NGS for 1 h, and then for 24 h with primary antibody, anti-V5 tag antibody (1:2,000, Thermo Fisher Scientific, Cat No. 46-0705), and anti–α-tubulin (YL1/2, 1:500, Santa Cruz Biotechnology, Cat No. sc53029) diluted in PBST at 4°C. Cells were washed in PBST and then incubated for 1 h with secondary antibody solution containing Alexa Fluor 488 goat anti–mouse IgG (H + L) (1:1,000; Thermo Fisher Scientific, Cat No. A11029), Alexa Fluor 568 goat anti–rat IgG (H + L) (1:1,000; Thermo Fisher Scientific, Cat No. 11007), and Hoechst 33342 (1:5,000; Thermo Fisher Scientific, Cat No. H3570) in PBST. Cells were washed in PBS and then mounted using ProLong Gold Antifade Reagent (Thermo Fisher Scientific, Cat No. P36934). The cells were imaged using *a BZ-X800 microscope* (Keyence). Neurite length was measured using the Neuroanatomy plugin of ImageJ software.

### Quantification and statistical analysis of axon length in Neuro2a cells

Immunostained Neuro2a cells were examined using a BZ-X800 microscope (Keyence) under ×20 magnification. V5-tagged CRMP1 (WT, Thr509Asp mutant) was visualized with the Alexa Fluor 488 channel, tubulin with the Alexa Fluor 568 channel, and cell nuclei with Hoechst 33342. Neurite length analysis was performed using the Neuroanatomy plugin of ImageJ software. Differences between groups were analyzed with the 2-sided *t*-test. All statistical analyses were performed using GraphPad Prism software (v 8.4.3). *p* values < 0.05 were considered statistically significant.

## Results

### Specificity of anti–pThr509-CRMP1 antibody

**Figure 1:**
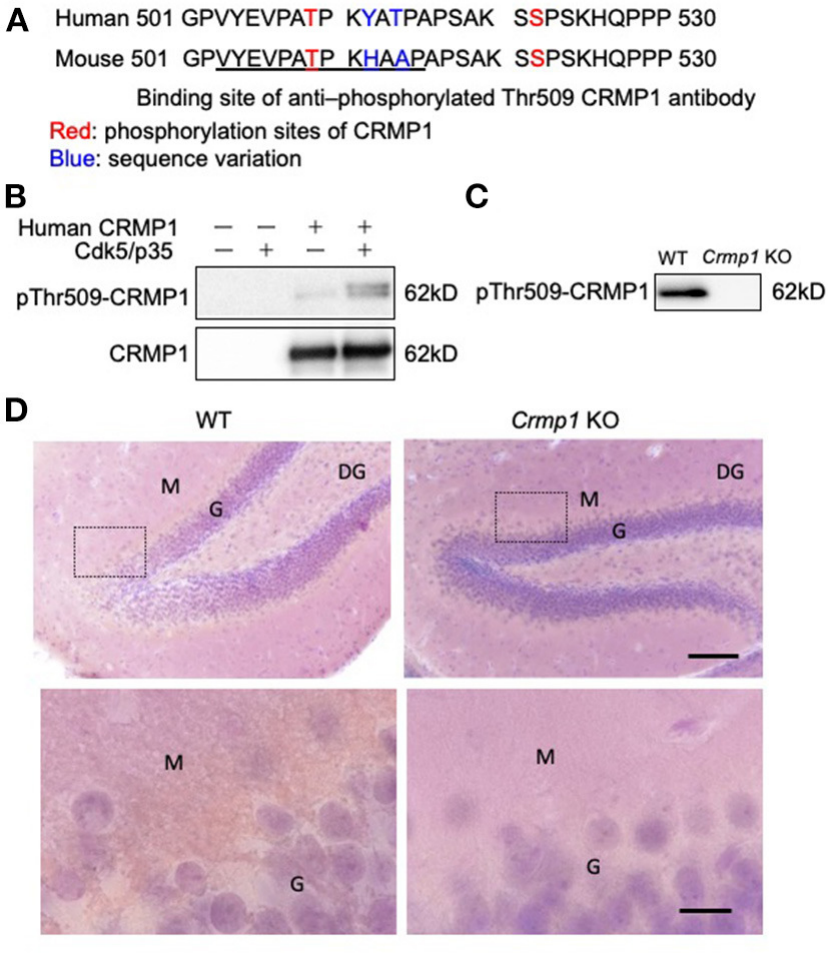
Specificity of anti–pThr509-CRMP1 antibody. **(A)** Sequence comparison of the C-terminal domain in human and mouse CRMP1. Underlined letters indicate the antigen peptides comprising the anti–pThr509-CRMP1 antibody. **(B)** Phosphorylation of Thr509 in CRMP1 by Cdk5. HEK 293T cells were transfected with human CRMP1 and Cdk5. The anti–pThr509-CRMP1 antibody successfully recognized pThr509 in human CRMP1. **(C)** Immunoblotting of brain lysates of WT and Crmp1 knockout KO mice with anti–pThr509-CRMP1 antibody. pThr509-CRMP1 signals are lost in Crmp1 KO mice. **(D)** DAB labeling of hippocampi of WT and Crmp1 KO mice with an anti–pThr509-CRMP1 antibody. The lower panels show enlargement of the area enclosed by the dotted line in the upper panels. Brown DAB chromogen is observed in the dendrite-rich molecular layer of the hippocampal dentate gyrus in WT mice, but is not seen in Crmp1 KO mice. Scale bars = 100 μm (top); 20 μm (bottom). DG, dentate gyrus; G, granule cell layer; M, molecular layer.

To specifically investigate the phosphorylation status of CRMP1 in ALS spinal cords, we used anti–pThr509-CRMP1 antibody raised against mouse/rat CRMP1 (503–515) peptide (Figure 1A) instead of the commonly used phospho-antibody against Ser522-CRMP1/2, which is an identical phosphorylation consensus motif for CRMP1 and CRMP2. The reactivity of our antibody with human CRMP1 was verified by western blotting using HEK 293T cell lysate transfected with human CRMP1, either with or without exogenous Cdk5 expression (Figure 1B). We found that the antibody reacted with the phosphorylated human CRMP1 under the presence of Cdk5, indicating that Cdk5 phosphorylates Thr509 of CRMP1. Using the brain tissues of *Crmp1* knockout mice, the specificity of this antibody for CRMP1 was further confirmed by negative results with immunoblotting of brain lysate (Figure 1C). pThr509-CRMP1 immunoreactivity was observed in areas corresponding to the dendrite-rich molecular layer of the hippocampal dentate gyrus in WT mice, but not in CRMP-1KO mice (Figure 1D).

### Accumulation of pThr509-CRMP1 in the lumbar spinal cord of ALS patients

**Figure 2:**
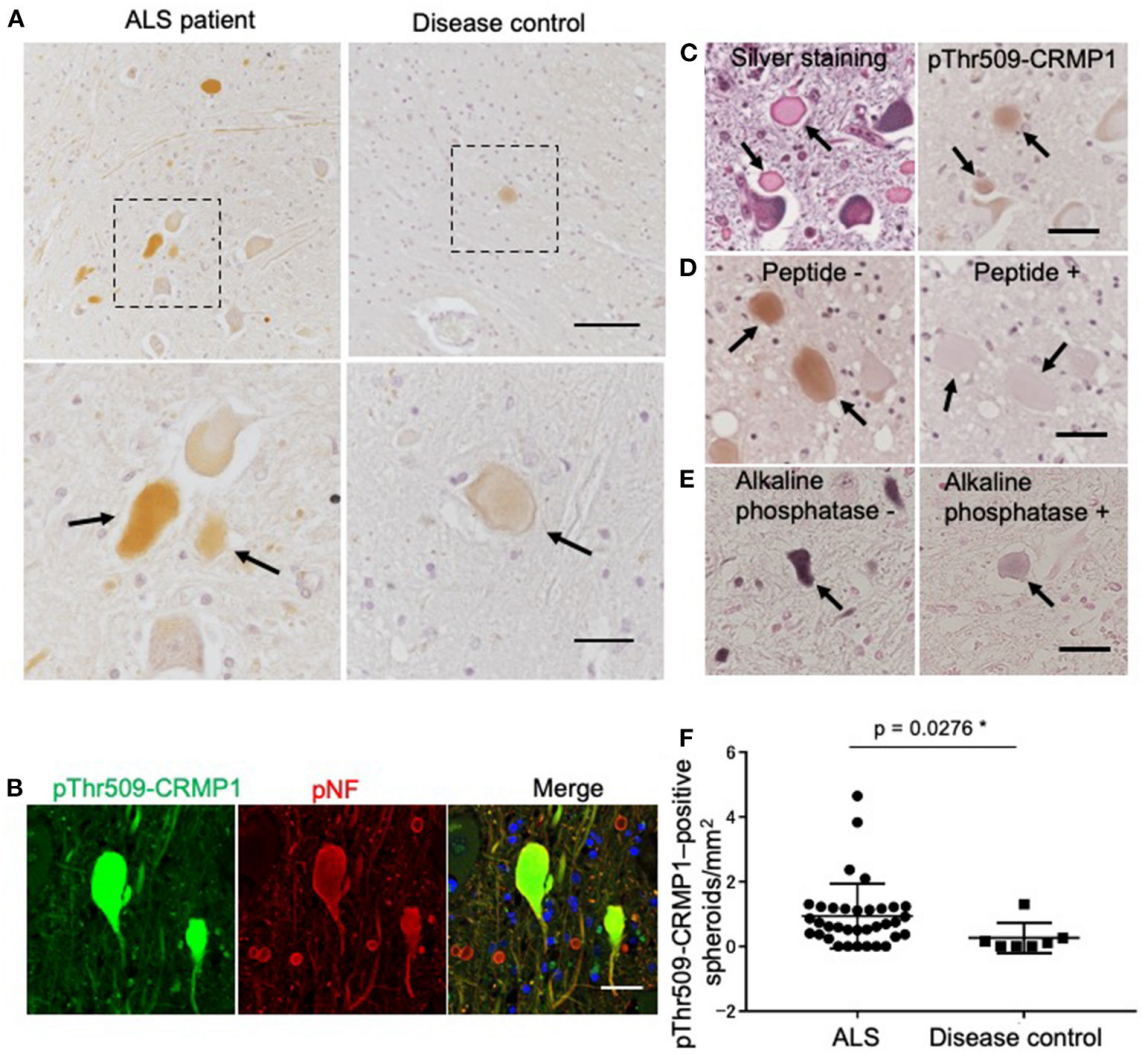
pThr509-CRMP1 staining in lumbar spinal cords of ALS patients. **(A)** Immunostaining of pThr509-CRMP1 in lumbar spinal cords of patients with ALS (left) and disease controls (right). There were many pThr509-CRMP1–positive oval or round structures (arrows) in ALS patients but rarely in disease controls. The bottom figures show enlarged views of the enclosed squares in the top figures. Scale bars = 100 μm (top); 30 μm (bottom). **(B)** Double immunofluorescence labeling of pThr509-CRMP1 and pNFs, the latter of which are a major component of spheroids. Co-localization of both proteins indicated that pThr509-CRMP1 is also a component of spheroids. Scale bar = 50 μm. **(C)** Bodian silver staining and pThr509-CRMP1 immunostaining of consecutive sections in ALS spinal cords. Spheroids are positive for both stains. Scale bar = 50 μm. **(D)** Although the epitope of the anti–pThr509-CRMP1 antibody was derived from a mouse sequence, a phosphopeptide with a human sequence effectively blocked the immunostaining signals of spheroids. Scale bar = 50 μm. **(E)** Alkaline phosphatase treatment attenuated pThr509-CRMP1–positive signals of spheroids. Scale bar = 50 μm. **(F)** The number of pThr509-CRMP1–positive spheroids per anterior horn area (mm^2^) in ALS patients and disease controls. *p < 0.05.

We examined the phosphorylation status of CRMP1 in the lumbar spinal cord of ALS patients by immunohistochemistry with the anti–pThr509-CRMP1 antibody, and detected immunoreactivity in ALS patients but rarely in disease controls (Figure 2A). The observed oval or round immunostaining pattern resembled the morphology of spinal spheroids. In fact, these pThr509-CRMP1–positive structures co-immunostained with pNFs, a major cytopathological hallmark of spheroids (Figure 2B), in most of the examined pThr509-CRMP1–positive structures. These structures were also positive for Bodian silver staining in consecutive sections (Figure 2C). To further characterize the pThr509-CRMP1 signal in ALS tissues, we performed peptide block and dephosphorylation assays. As shown in Figures 2D,E, a human sequence–derived CRMP1 (503–515) peptide and also alkaline phosphatase blocked immunostaining of pThr509-CRMP1. We therefore concluded that pThr509-CRMP1 is a constituent of spheroids in ALS spinal cords.

Next, we performed pThr509-CRMP1 immunostaining of lumbar spinal cords in 35 patients with ALS and seven disease controls (Table 1), and calculated the number of pThr509-CRMP1–positive spheroids in each patient along with the remaining motor neurons and the anterior horn area. The number of pThr509-CRMP1–positive spheroids per anterior horn area (mm^2^) in the ALS patient group was significantly higher than that in the disease control group (Figure 2F) and was negatively correlated with disease duration, but there was no correlation with age at death, initial symptoms, walking score of the ALS Functional Rating Scale–Revised, or the number of residual neurons (Supplementary Table S2). The statistical methods used are listed in Supplementary Table S2.

### Accumulation of pThr509-CRMP1 precedes that of pNFs in spheroids of ALS patients

**Figure 3:**
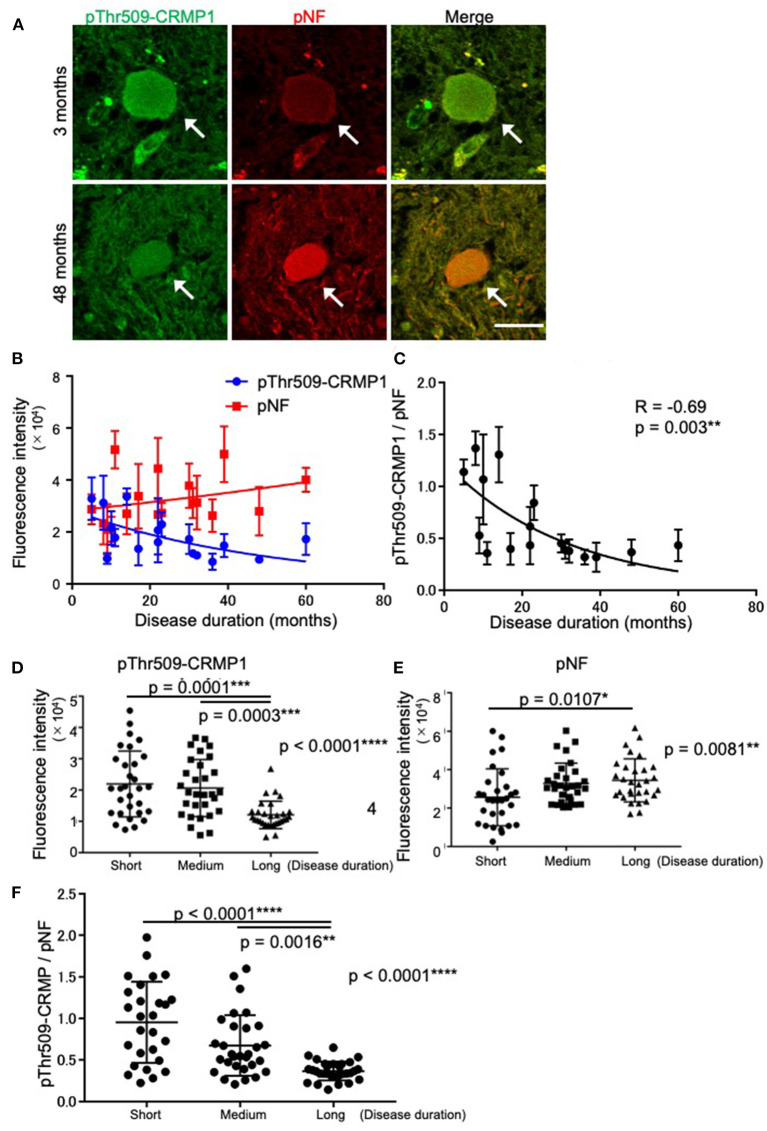
Time-dependent expression relationships between pThr509-CRMP1 and pNFs in spinal spheroids. **(A)** Double immunofluorescence labeling of spinal spheroids with anti–pThr509-CRMP1 antibody (green) and anti-pNF antibody (red) in two ALS patients with short (3 months) or long (48 months) disease duration. Scale bar = 30 μm. **(B)** Mean fluorescence intensity of pThr509-CRMP1 (blue) and pNFs (red) in spheroids of the two ALS patients with a different disease duration (*n* = 18). **(C)** The pThr509-CRMP1: pNF fluorescence ratio in spheroids was significantly and negatively correlated with disease duration. **(D–F)** The fluorescence values of pThr509-CRMP1 **(D)** and pNFs **(E)**, and the pThr509-CRMP1: pNF ratio **(F)** in each spheroid, in 18 ALS patients according to short (7.67 ± 3.08 (3–11) months), medium (21.3 ± 5.50 (14–30) months), and long (41.0 ± 11.0 (31–61) months) disease duration. *p < 0.05, **p < 0.01, ***p < 0.001, ****p < 0.0001.

While verifying the co-localization of pThr509-CRMP1 and pNFs in spheroids, we noticed that the immunofluorescence intensity of pThr509-CRMP1 was higher than that of pNFs in the spinal cord of an ALS patient with short disease duration (3 months), and vice versa in a patient with a long disease duration (48 months) (Figure 3A). Therefore, we investigated the effect of disease duration on the fluorescence intensity of pThr509-CRMP1 and pNFs in 18 evaluable patients with ALS (Figure 3B). As the disease progressed, the mean fluorescence intensity of pThr509-CRMP1 in each patient decreased, while that of pNFs increased. The pThr509-CRMP1:pNF fluorescence intensity ratio was negatively correlated with disease duration (Figure 3C). We also plotted the fluorescence values of pThr509-CRMP1 (Figure 3D) and pNFs (Figure 3E), and the pThr509-CRMP1:pNF ratio (Figure 3F) in all spheroids according to short, medium, and long disease durations, and demonstrated statistical differences between the three duration groups. These results suggest that accumulation of pThr509-CRMP1 may precede that of pNFs in ALS spheroids.

### Inhibition of neurite outgrowth by the phosphomimic form of CRMP1 in Neuro2a cells

**Figure 4:**
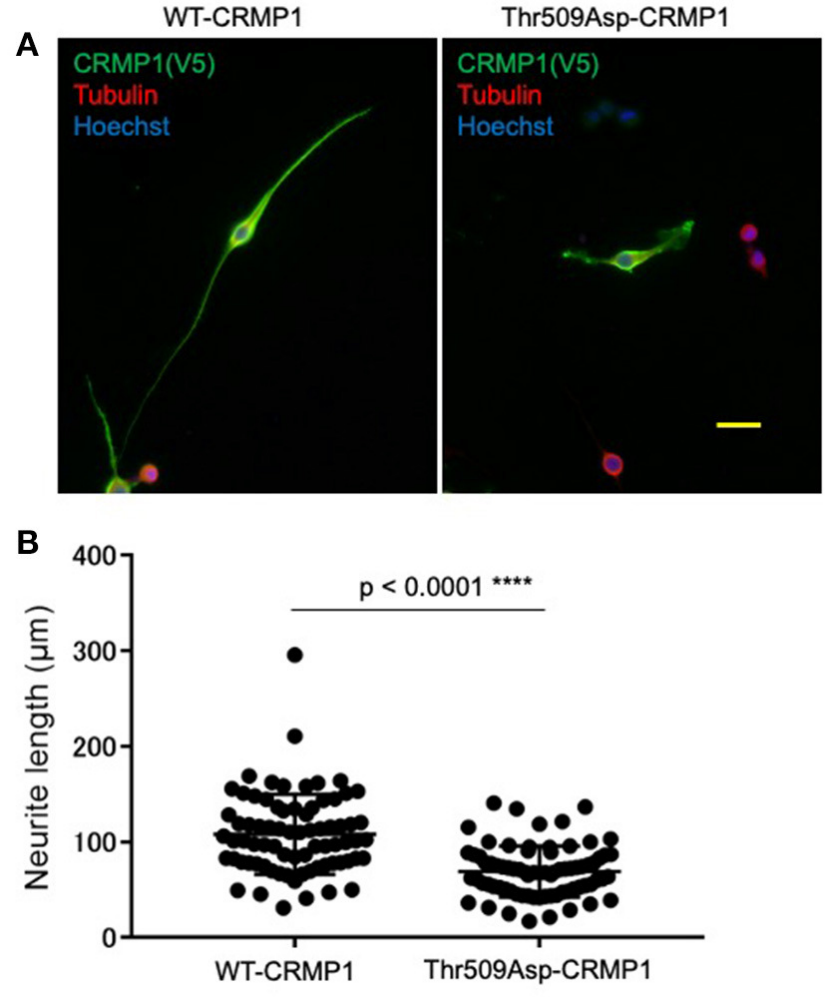
Effect of CRMP1 phosphorylation on neurite outgrowth. **(A)** Representative images of neurite elongation in Neuro2A cells transfected with either WT-CRMP1 (left) or the phospho-mimicking CRMP1 mutant Thr509Asp-CRMP1 (right). Scale bar = 50 μm. **(B)** Quantitative analysis of neurite lengths. The five longest neurites were assessed in each of five fields using ImageJ software. The experiments were repeated three times and a total of 75 neurites were evaluated in each transfection. ****p < 0.0001.

To clarify the potential role of Thr509 phosphorylation in neuron CRMP1, we examined how the overexpression of the phospho-mimicking CRMP1 mutant Thr509Asp-CRMP1 affected neurite outgrowth in murine neuroblastoma Neuro2a cells, which are commonly used to study neuronal cytoskeletal dynamics (29). Neuro2a cells were transfected with an expression vector harboring either V5-tagged WT- or Thr509Asp-CRMP1. The cells were differentiated by a cAMP analog to facilitate neurite outgrowth. We found that Thr509Asp-CRMP1–expressing cells had shorter neurites than WT-CRMP1–expressing cells (Figure 4). These data suggest that the phosphorylation of CRMP1 at residue Thr509 may inhibit neurite outgrowth.

## Discussion

In this study, we demonstrated that pThr509-CRMP1 accumulates in the spheroids of human ALS motor neuron axons before pNFs, suggesting an early contribution of pThr509-CRMP1 to spheroid pathology. Taken together with the inhibition of neurite outgrowth by pThr509-mimicking CRMP1, it is plausible that pCRMP1 is involved in the pathogenesis of ALS.

CRMP1 is a member of the CRMP family of proteins, which are involved in signaling downstream of Sema3A, a repulsive axon guidance molecule; CRMP1 also regulates neuronal migration, dendritic spine development, and synaptic plasticity (17, 30, 31). Sema3A signaling stimulates phosphorylation of CRMP1/2 *via* Src family Fyn tyrosine kinase and Cdk5 (15–17, 19). In CRMP1 and CRMP2, it has been reported that C-terminal Ser522 priming by Cdk5 is followed by sequential phosphorylation of Ser518, Thr514, and Thr509 by GSK3β (18, 32, 33), but some steps in this process on CRMP1 are still unconfirmed. In this study, an anti–pThr509-CRMP1 antibody was raised against the mouse/rat CRMP1 peptide (Figure 1A), and Thr509 in CRMP1 is reported to be directly phosphorylated by Cdk5 in rodents (33). However, it remains uncertain whether human pThr509-CRMP1 reacts with this antibody and is directly phosphorylated by Cdk5. As shown in Figure 1B, we demonstrated that anti–pThr509-CRMP1 antibody successfully detected human pCRMP1, and Cdk5 phosphorylated CRMP1 in human cells (HEK 293T) without the need for GSK3β. Furthermore, CRMP1 specificity was confirmed using brain tissues of *Crmp1* KO mice (Figures 1C,D).

It has been suggested that CRMP1 is involved in the pathogenesis of neurological diseases such as Huntington's disease (34) and schizophrenia (35, 36). Although its contribution in ALS had not been elucidated except that mass spectrometry data suggests CRMP1 is one of the interacting partners of the Met337Val TDP-43 mutant (37), we recently have shown that CRMP1 modulates phenotypes of G93A-SOD1 mice (23).

In this study, we investigated the localization of pCRMP1 in the spinal cord of ALS patients by immunostaining using CRMP1-specific anti-pThr509 antibody. Remarkably, pThr509-CRMP1 was found at the sites of motor neuron axon swelling but was not clearly observed in the cell bodies (Figure 2A). The large hydrophilic axonal swellings in the proximal axons are known as spheroids, and they consist of accumulated pNFs, peripherin, mitochondria, and lysosomes (38–40). pThr509-CRMP1 co-localized with pNFs in our study (Figure 2B), indicating that the oval or round structures in the ALS spinal cords were spheroids. Axonal spheroids are not specific to ALS, and small numbers of spheroids may also appear with increasing age in healthy elderly individuals and non-ALS neurological patients. We found that pThr509-CRMP1–positive spheroids were most common in ALS patients (Table 1; Figure 2F), but were also rarely seen in disease controls (Figure 2A), suggesting that pThr509-CRMP1 accumulation in spheroids is not a phenomenon unique to ALS.

Among various neurofilament subunits, neurofilament medium chain (NF-M), and neurofilament heavy chain (NF-H) demonstrate particularly high phosphorylation levels in axons in pathological conditions such as ALS. This phosphorylation is considered to slow the axonal transport of pNFs (41, 42), resulting in their accumulation and the formation of spheroids. pNF accumulation also leads to a further delay in anterograde axonal transport, as shown in an ALS mouse model (43). Spheroids in ALS patients are also immunoreactive against galectin-1 (44), and galectin-1 promotes the aggregation of NFs through its ability to bind and cross-link various molecules (45). Taken together, spheroid proteins may be involved in the pathogenesis of ALS, either as a cause, facilitator, or consequence of axonal transport dysfunction, by interacting with each other.

Interestingly, our results from 18 ALS patients with various disease durations indicated that pThr509-CRMP1 was one of the spheroid proteins that accumulated prior to pNFs (Figure 3). We speculate that this is because axonal dysfunction caused by pThr509-CRMP1 leads to axonal transport deficits, which in turn induces prior accumulation of pThr509-CRMP1 and subsequent accumulation of pNFs. Indeed, our *in vitro* experiment showed that the phospho-mimicking mutant of CRMP1, Thr509Asp-CRMP1, caused distal axonal dysfunction as shown by axonal outgrowth inhibition that was presumably caused by growth cone collapse at the distal ends of neurites (Figure 4). Distal axonal dysfunction through Sema3A–CRMP signaling has also been suggested to occur in ALS mice. Sema3A is highly expressed in Schwann cells at the distal ends of motor neuron axons in G93A-SOD1 ALS mice (20). Furthermore, blockade of Sema3A signaling by anti-NRP1 antibody (21) or inhibition of CRMP1 phosphorylation (23) mitigates NMJ damage and restores muscle strength in the G93A-SOD1 mouse model of ALS. These findings are consistent with ALS pathogenesis, which is characterized by progressive distal axonopathy in motor neurons that precedes degeneration of cell bodies (10–13).

In ALS patients, distal axonopathy caused by phosphorylation of CRMP1/2 is speculated to trigger dysfunction of intracellular axonal transport, with damage extending to proximal axons (forming spheroids) and then to the cell body. Phosphorylation of CRMP2 is known to cause axonal transport dysfunction by inhibiting CRMP2 binding to tubulin dimers and to the anterograde motor protein, kinesin light chain (46–48). It has also been shown that the kinesin-1 adaptor fasciculation and elongation protein zeta 1 (FEZ1), which is involved in axonal transport, forms a complex with CRMP1 in axons and nerve growth cones (49). The effect of CRMP1 phosphorylation on its binding to FEZ1 and axonal transport is currently unknown. However, considering the established evidence regarding axonal transport dysfunction upon CRMP2 phosphorylation (47, 48) and CRMP1 involvement in axonal transport (50), it is likely that pThr509-CRMP1 also affects transport function and contributes to its own accumulation of pThr509-CRMP1 in proximal axons as spheroids.

Recent studies have reported that phosphorylated TDP-43 (pTDP-43) accumulates and interferes with synaptic protein synthesis in axons and NMJs of ALS patients, and this subsequently induces neurodegeneration (51, 52). Regarding the relationship between CRMP1 and TDP-43, non-phosphorylated CRMP1 suppresses TDP-43 aggregation (34), and CRMP1 interacts with TDP-43 (35). Therefore, excessive phosphorylation of CRMP1 at the distal ends of axons may inhibit neurite outgrowth (Figure 4) and contribute to NMJ pathogenesis in ALS, together with TDP-43, such as by affecting axonal pTDP-43 aggregation. However, this speculation requires further investigation.

Finally, spheroid proteins are among the most promising candidate biomarkers for ALS, as exemplified by pNF-H (53–56) and peripherin (57). As for the application of CRMPs, the pCRMP2:CRMP2 ratio in lymphocytes was recently reported to be a useful biomarker for schizophrenia (58). Because CRMPs are involved in the maintenance of the cytoskeleton, along with neurofilaments and peripherin, and accumulation of pThr509-CRMP1 precedes that of pNFs, it is worthwhile to investigate pCRMP1 as a possible early biomarker for ALS.

One limitation of our study is that differences in mean fluorescence intensity were used to demonstrate that accumulation of pThr509-CRMP1 precedes that of pNFs in ALS spheroids, given that most of the examined spheroids larger than 20 μm were already double positive for pThr509-CRMP1/pNFs. If we could show the number of pThr509-CRMP1–positive/pNF-negative spheroids was much higher than that of pNF-positive spheroids in ALS cases with short duration, our conclusion might have been more convincing. pThr509-CRMP1–positive/pNF-negative staining may be present in swollen axons smaller than 20 μm.

**Figure 5:**
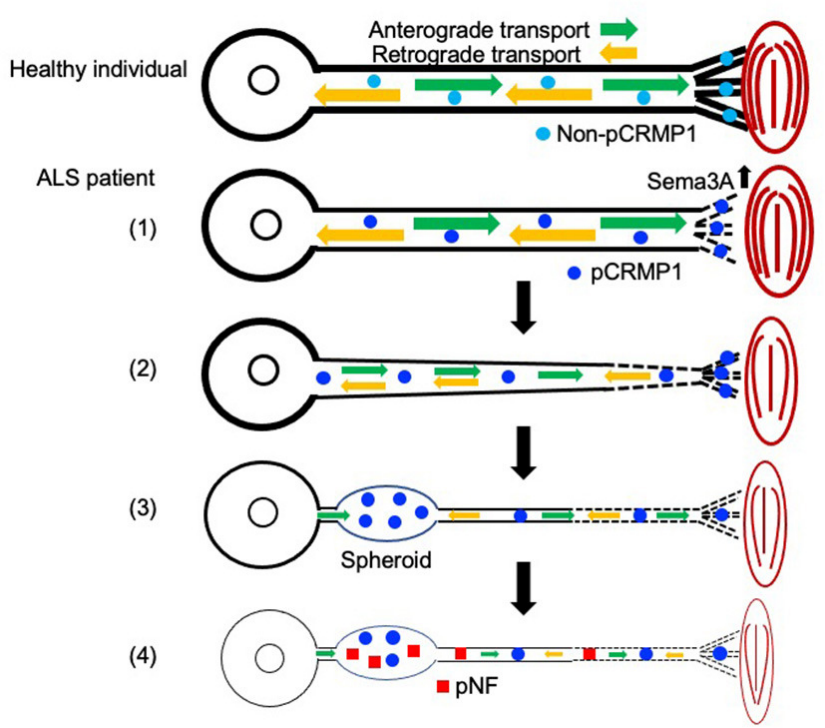
Schematic diagram showing the hypothetical mechanism by which axonal function in ALS is influenced by CRMP1 phosphorylation mediated by Sema3A–Cdk5 signaling. Non-phosphorylated CRMP1 in healthy individuals maintains the NMJ and contributes to axon elongation by virtue of the preserved function of the distal axon (see Figure 4). In ALS, (1) enhanced Sema3A signaling in the NMJ leads to CRMP1 phosphorylation by Cdk5, and this phosphorylation is responsible for aspects of distal axonal dysfunction such as NMJ denervation and inhibition of axonal outgrowth (see Figure 4); (2) pCRMP1 mediates the progression of distal-to-proximal axonopathy (dying back), at least partly through axonal transport dysfunction; (3) pCRMP1 accumulates as spheroids in proximal axons due to transport dysfunction; and (4) pNF accumulation is promoted by axonal transport dysfunction and the presence of pCRMP1 as an obstacle.

In this study, we first showed that pCRMP1 is one of the components of spheroids in ALS patients and that pCRMP1 accumulation may precede the accumulation of pNFs. Our hypothesis regarding pCRMP1 involvement in ALS pathogenesis is as follows (Figure 5): (1) enhanced Sema3A signaling in NMJs leads to Thr509-CRMP1 phosphorylation by Cdk5, and this phosphorylation is responsible for aspects of distal axonal dysfunction such as NMJ denervation and inhibition of axonal outgrowth; (2) pCRMP1 mediates the progression of distal-to-proximal axonopathy (dying back), at least partly through axonal transport dysfunction; (3) pCRMP1 accumulates as spheroids in proximal axons due to transport dysfunction; and (4) pNF accumulation is promoted by axonal transport dysfunction and the prior accumulation of pCRMP1 as a physical obstacle to transport. To prove this hypothesis, it is necessary to assess the phosphorylation status of CRMP1 in distal axons in ALS patients and to clarify the temporal relationship between pCRMP1 and pNF accumulation through experiments involving both mouse and cell models. Furthermore, the effect of phosphorylating CRMP1 on axonal transport need to be addressed.

## Data availability statement

The raw data supporting the conclusions of this article will be made available by the authors, without undue reservation.

## Ethics statement

The animal study was reviewed and approved by the Institutional Review Board of Yokohama City University School of Medicine. The studies involving human participants were reviewed and approved by the Institutional Review Board of Yokohama City University School of Medicine (B090903014). The patients/participants provided their written informed consent to participate in this study.

## Author contributions

YK and FT designed the experiments. YK co-ordinated the study with the guidance of FN, YG, and FT. YK and MT performed neuropathology experiments. YK and FN performed the cell biology experiments. TO generated the anti–pThr509-CRMP1 antibody. TA, HN, AJ-T, HM, SK, and SH prepared experimental materials. YG, HT, HD, FN, and FT supervised the experiments. YK, FN, and FT drafted the manuscript. All authors read and approved the final manuscript.

## Funding

This study was supported by Grants-in-Aid for Scientific Research from the Japan Society for the Promotion of Science (JSPS) (#18K15457 to HN; #17082006 to YG; and #18K07532 to FT), Targeted Proteins Research Program from JSPS Global COE Program (#0761890004 to YG), Creation of Innovation Centers for Advanced Interdisciplinary Research Areas Program in the Project for Developing Innovation Systems from the Ministry of Education, Culture, Sports, Science and Technology (MEXT) (#42890001 to YG), Health Labor Sciences Research Grants from the Ministry of Health, Labor and Welfare (#202011073A and 202011029A to FT), the Wakaba Research Fund (Research Fund for Potential Young Researchers to HN) from the Yokohama Foundation for Advanced Medical Science, and a Grant for Strategic Research Promotion from Yokohama City University (SK2804 to FT).

## Conflict of interest

The authors declare that the research was conducted in the absence of any commercial or financial relationships that could be construed as a potential conflict of interest.

## Publisher's note

All claims expressed in this article are solely those of the authors and do not necessarily represent those of their affiliated organizations, or those of the publisher, the editors and the reviewers. Any product that may be evaluated in this article, or claim that may be made by its manufacturer, is not guaranteed or endorsed by the publisher.
