# Supplementary material for: Phosphorylated CRMP1, axon guidance protein, is a component of spheroids and is involved in axonal pathology in amyotrophic lateral sclerosis
Source: Front Neurol. 2022 Sep 27;13:994676. doi: 10.3389/fneur.2022.994676 (PMC9552802; doi:10.3389/fneur.2022.994676)
Supplement: Supplementary file 1 [file Data_Sheet_1.docx]

Supplementary Material

| Antibody (mAb clone) | Vendor, (Catalog #), RRIDs, | Dilution | Application |
| --- | --- | --- | --- |
| pThr509-CRMP1, rabbit pAb | (Created by Prof. Ohshima) | 1:1000 | IB |
|  |  | 1:300 | IHC |
| pNF, mouse mAb | BioLegend, (SMI 31P), RRID: AB_2564641 | 1:1000 | IHC |
| V5, mouse mAb | Invitrogen, (R960−25), RRID: AB_2556564 | 1:2000 | ICC |
| α-tubulin (YL1/2), rat mAb | Santa Cruz Biotechnology, (sc-53029), RRID: AB_793541 | 1:500 | ICC |
| pTDP-43S409/410, mouse mAb | CosmoBio, (TIP-PTD-M01), RRID: AB_1961900 | 1:1000 | IHC |
| FUS | Sigma, (HPA008784), RRID: AB_1849181 | 1:1000 | IHC |
| Polyclonal goat anti–rabbit immunoglobulins/biotinylated | Agilent, (E0432), RRID: AB_2313609 | 1:1000 | IHC |
| Alexa Fluor 488–labeled goat anti–mouse IgG | Invitrogen, (A11029), RRID: AB_ 2534088 | 1:1000 | ICC |
| Alexa Fluor 488–labeled goat anti–rabbit IgG | Invitrogen, (A11034), RRID: AB_2576217 | 1:1000 | IHC |
| Alexa Fluor 594–labeled goat anti–mouse IgG | Invitrogen, (A11032), RRID: AB_2534091 | 1:1000 | IHC |
| Alexa Fluor 594–labeled goat anti–rat IgG | Invitrogen, (A11007) , RRID: AB_141374 | 1:1000 | ICC |
| Hoechst 33342 | Thermo Fisher Scientific, (H1399), RRID: AB_2534069 | 1:5000 | IHC, ICC |

**Supplementary Table 1. Antibody list**

Abbreviations: mAb, monoclonal antibody; pAb, polyclonal antibody; pNF, phosphorylated neurofilament; IB, immunoblotting; ICC, immunocytochemistry; IHC, immunohistochemistry; RRID, research resource identifier

| Factor | Analysis method | r | 95% CI | p value |
| --- | --- | --- | --- | --- |
| Age at death | Spearman | −0.1113 | −0.437 to 0.2402 | 0.5245 |
| Disease duration | Spearman | −0.4312 | −0.6741 to 0.1043 | 0.0097 |
| Initial site of symptom | Kruskal-Wallis |  |  | 0.6552 |
| Walking score (ALSFRS-R) | Kruskal-Wallis |  |  | 0.5183 |
| No. of residual neurons | Spearman | 0.3221 | 0.2271– 0.5984 | 0.0591 |

**Supplementary Table 2. Correlation analysis of the pThr509-CRMP1­–positive spheroids in ALS patients**

Abbreviations: CI, Confidence Interval; ALSFRS-R, ALS Functional Rating Scale–Revised; Spearman, Spearman’s rank correlation coefficient; Kruskal-Wallis, Kruskal-Wallis test
